# Impact of COVID-19 on continuing professional development: Perspectives of audiologists

**DOI:** 10.4102/safp.v66i1.5963

**Published:** 2024-08-07

**Authors:** Suvishka Barath, Andrew J. Ross

**Affiliations:** 1 Department of Family Medicine, College of Health Sciences, University of KwaZulu-Natal, Durban, South Africa

**Keywords:** pandemic, COVID-19, online learning, hybrid learning, young audiologists, private sector, continuing professional development

## Abstract

**Background:**

The coronavirus disease 2019 (COVID-19) pandemic triggered unprecedented disruptions to continuing professional development (CPD) activities, which are essential for healthcare professionals (HCPs) to stay abreast on best practices, current knowledge and emerging technologies, ultimately enhancing patient care. Audiologists encountered multiple challenges during the pandemic, necessitating adaptations and innovations in their CPD practices. While literature was published during the pandemic on shifting education systems to online platforms, little is known about its impact on the CPD of young audiologists working in the private sector.

**Methods:**

A descriptive, qualitative research design was adopted to collect rich data from 11 audiologists using online semi-structured interviews which were thematically analysed using Braun and Clark’s steps.

**Results:**

COVID-19 brought about both positive adaptations and negative disruptions to the CPD activities of young audiologists. Eight major themes were identified in this study. These include (1) the adoption of online learning, (2) improved flexibility, (3) cost-effectiveness, (4) diverse learning opportunities, (5) keeping current, (6) isolation and networking, (7) limited interactivity and (8) uncertain quality assurance.

**Conclusion:**

The COVID-19 pandemic had a considerable influence on the CPD activities of young audiologists in the private sector. While presenting significant challenges, including disruptions to traditional learning modalities, the pandemic also catalysed innovation and adaptation within the profession.

**Contribution:**

This study highlights the resilience exhibited by young audiologists towards their CPD and also provides actionable insights for informing professional development initiatives, tailored to the evolving needs of audiologists in the post-COVID-19 era.

## Introduction

On 11 March 2020, the World Health Organization (WHO) declared the coronavirus disease 2019 (COVID-19) as a global pandemic,^1^ which brought unprecedented challenges to healthcare systems worldwide, profoundly impacting the professional landscape of healthcare professionals (HCPs).^2^ Pandemic restrictions were imposed across South Africa on 23 March 2020, the 6 weeks of stay-at-home being followed by 15 months of varying levels of travel and contact limitations. This not only had implications for people needing to access health services but also for education across the country, including for HCPs. In South Africa, HCPs are required to undertake continuing professional development (CPD) activities annually as part of the requirement to maintain their membership with the Health Professionals Council of South Africa (HPCSA), which is their representative statutory body. Continuing professional development, which is integral to maintaining and enhancing the competence of HCPs, encountered both disruptions and innovations in response to the pandemic.^3^ The CPD of HCPs experienced significant transformations because of the increased demand for CPD opportunities to be tailored to the pandemic response,^2^ that being the inability to meet in person. While the progress made in combating the virus and the gradual return to normality is celebrated, it is important to reflect on the impact the pandemic had on the CPD of young audiologists.

As healthcare systems grappled with surges in COVID-19 cases, HCPs faced shifting priorities, increasing resource constraints and the need for rapid adaptation to evolving clinical practices.^4^ Consequently, the traditional modes of CPD delivery, such as in-person conferences and workshops, underwent substantial modifications to align with public health measures, such as social distancing, to be able to address the emergent needs of HCPs.^2^ With restrictions on travel and in-person gatherings, there was a significant increase in virtual CPD activities.^5^ Webinars, online courses, virtual conferences and remote workshops became the norm, allowing HCPs to continue their learning while adhering to the pandemic requirements.^2^ Although the restrictions imposed by COVID-19 were lifted in July 2022, many CPD activities have continued online or in a hybrid format. Online learning involves educational activities conducted via the internet, utilising synchronous or asynchronous methods that can be accessible through a range of internet-connected devices.^6^ However, hybrid learning is an educational approach that integrates traditional workplace activities with enhanced online environments, leveraging technology to create a blended learning experience.^7^

Studies have been conducted to assess the effectiveness of online learning during the pandemic, and present conflicting findings, many having taken place at its outset, thus rendering them inapplicable to the current professional development of HCPs in the post-pandemic era.^8,9,10,11^ Currently, there is insufficient literature to rigorously evaluate the impact of this considerable change in the provision of CPD and its influence on the practice of HCPs. This is pertinent, as CPD is arguably the most important aspect of healthcare education across healthcare professions after graduation.

Although the COVID-19 pandemic limited access to and the provision of healthcare services, it is important to understand the professional development of HCPs who graduated amid the pandemic, as there are invaluable lessons to be learned from this unparalleled experience that may shape the future of healthcare education, practice and preparedness. Gaining insight into audiologists’ experience of how COVID-19 impacted their CPD may assist the providers in reshaping these activities. This would allow CPD providers to embrace these shifts and utilise innovative approaches, and enable audiologists to successfully navigate the challenges of accessing CPD and maintaining high standards of patient care in the post-pandemic period. Therefore, this study aimed to explore the impact of COVID-19 on the CPD activities of young audiologists working in the South African private sector.

## Research methods and design

This study employed a descriptive qualitative research design involving online semi-structured interviews with 11 audiologists employed in the private sector in KwaZulu-Natal (KZN), South Africa. The methods employed in this study closely follow those detailed in the author’s previously published article titled ‘Conceptualising the experiences of continuing professional development of young private sector audiologists as an attribute of andragogy’.^12^

Purposive sampling was utilised, starting with the distribution of a participant recruitment poster across KZN using various social media platforms targeting audiologists. Prospective participants contacted the researcher to express interest with snowball sampling subsequently being employed to acquire referrals to additional audiologists. Interested audiologists received an information document via email or WhatsApp to aid with their decision-making process. Eligible participants who agreed to participate completed informed consent forms prior to engaging in semi-structured interviews through WhatsApp voice calls, which lasted between 20 min and 40 min. The researcher utilised an interview schedule based on emerging themes in literature to guide the semi-structured interview (Appendix 1). Following data collection, verbatim transcriptions of the interviews were conducted from audio recordings. The data were then deductively analysed using Braun and Clarke’s six-step thematic analysis process.^13^

A pilot study involving two participants was conducted to validate and refine the data collection tool for credibility and efficiency. To enhance data reliability and mitigate bias, an independent qualitative coder was engaged. After independently identifying codes, categories and themes, a consensus meeting was convened between the researcher and the independent coder to discuss and finalise the coding framework and thematic analysis.

### Ethical considerations

Ethical approval was obtained from the University of KwaZulu-Natal’s Humanities and Social Sciences Ethics Committee (HSSREC/00006281/2023).

## Results

Table 1 summarises the characteristics of the participants, all aged 23–26 years, most (*n* = 8) being female, and the majority (*n* = 10) having worked for less than 4 years. All 11 had engaged in online methods of CPD, with reading online journal articles being the most common activity and participating in online training courses the least.

**TABLE 1:** Description of participants.

Variable	Characteristic	*n*
Age (years)	23–24	6
	25–26	5
Gender	Female	8
	Male	3
Years worked in private practice	1–3	10
	4–5	1
Types of CPD activities	Reading online journal articles	11
	Participating in online training courses	3
	Attending webinars	4
	Joining in-person workshops	5

CPD, continuing professional development.

Eight major themes emerged from the study: the adoption of online learning, improved flexibility, cost-effectiveness, diverse learning opportunities, keeping current, isolation and networking, limited interactivity and uncertain quality assurance.

### The adoption of online learning

Participants indicated a drastic change in CPD in the wake of the COVID-19 pandemic, a shift still evident in their current activities. However, as they had not engaged in CPD before the pandemic, they could not make direct comparisons, but they do acknowledge the change brought about by COVID-19. Participants have observed a pronounced increase in online CPD activities, reflecting the broader shift towards online platforms:

‘I think most of the CPD moved online because of the COVID times, especially like those seminars which are now online.’ (Participant 4, Female, 23 years old)

‘I think COVID also impacted that [*CPD*] because everything just went online.’ (Participant 9, Female, 26 years old)

### Improved flexibility

Owing to the CPD activities moving online, participants indicated that they were able to access training materials and participate in courses at their convenience, particularly for asynchronous online CPD activities. This eliminated the need for travel and enabled them to balance their professional development with their work and personal responsibilities:

‘Since everything has now become more virtual and online based, there’s the advantage of convenience. So especially if you are someone that’s working in the private sector, it means you’re either working five to seven days a week. This means that you don’t always have the time to go out to workshops that are happening during the day, or you have to take leave from work. So online CPD has the added advantage of convenience, in that you can do it when you’re home or when you’ve got some spare time, so whenever you’re available.’ (Participant 6, Female, 25 years old)

### Cost-effectiveness

Participants indicated that CPD presented through virtual platforms was more affordable, as it eliminated the need to travel, making educational resources and training opportunities accessible to them, regardless of their location:

‘If they do have an in-person CPD you have to think of traveling costs because they might not have it in your areas. Online CPD is easier to access instead of actually attending in-person, because you were at risk of contracting COVID and even travelling costs, so I think it has benefits in a way you can access it from your home or your laptop or even your phone.’ (Participant 4, female, 23 years old)

### Diverse learning opportunities

Participants acknowledged that because of the lack of resources in their practices, they have limited exposure to high-end technology:

‘I think also with our country, we so limited because our country is not so developed in certain areas, for example vestibular. If you look at London they very good with those types of things, so with those areas it’s not so big here.’ (Participant 1, Female, 23 years old)

The online learning platforms allowed participants to access CPD activities at a global level, which enabled the young audiologists to engage with training programmes that best suited their learning needs and styles:

‘It also gives you access to professionals that are overseas. So with the virtual side, you have the advantage of attending CPD workshops that could be held by someone in America or the UK.’ (Participant 6, Female, 25 years old)

### Keeping current

Participants indicated that online CPD platforms assist in quickly disseminating information, which assisted them to stay abreast with the latest courses and research findings related to their practice. This ensured that they had access to the most current information to enhance their knowledge and skills:

‘In terms of lecturers abroad doing courses, especially upcoming courses on the advancements in technology, is great because it makes all of us aware as clinicians to involve best practices.’ (Participant 10, Female, 24 years old)

### Isolation and lack of networking

One of the drawbacks of online learning was the sense of isolation, as they missed the face-to-face interaction, the informal meeting opportunities, and the ability to network with peers and experts in their field and areas of interest:

‘[*online CPD*] … affects how you network, because if we had more in-person CPD activities, we’d be able to network more with other healthcare professionals and other audiologists in our areas. So with everything being online, you see people joining these webinars, but you don’t get to meet them in-person and discuss strategies, discuss new things that you’ve come across, new skills that you’ve learned and so in terms of networking, it does affect that you know.’ (Participant 6, Female, 25 years old)

Participants further highlighted that online CPD platforms feel impersonal, leading to a sense of disconnection from the learning experience and their colleagues. They indicated that participating in online CPD activities resulted in an absence of peer support, something that they would have benefitted from considering that they are relatively young professionals within the field of audiology:

‘I wish that there was more accessibility for in-person CPD programs, and I feel since COVID, we had all of this detachment in terms of networking. So, it’s a good way to collaborate by meeting in-person, especially after COVID, because I’ve graduated in the middle of COVID. So when I started my career, I didn’t have a network of audiologists that I knew and could contact, so if I was stuck with anything or I needed advice, it was basically just people I knew because we went to the same university or something. But I had a very limited network of healthcare co-workers that I could communicate with, which was definitely a disadvantage.’ (Participant 6, Female, 25 years old)

### Limited interactivity

While online CPD platforms offer flexibility, participants felt that such programmes lacked the interpersonal interaction and hands-on experiences that were provided by in-person training. Participants reported that this had an impact on both the depth of learning attained and their levels of engagement, which was negatively affected by the lack of familiarity with the other participants:

‘Because in an online platform, you don’t always have the courage to speak up if you have a certain difficulty with something, you’re less likely to ask questions online because it’s a bunch of people that you don’t really know or you haven’t met, but when you chat to people in-person and you get to know them, it’s easier to share struggles that you’re experiencing and it’s easier to say “how do you do this?” and “how do you overcome that?” and so that does make a difference as well.’ (Participant 6, Female, 26 years old)

### Uncertain quality assurance

Participants felt that the provision of activities through virtual platforms may be defeating the purpose of CPD, as they often witnessed their colleagues engaging to simply comply with the requirements of their statutory body, rather than using it as an opportunity to improve their knowledge and skills:

‘It’s one thing to learn all of the stuff online, but you do get a lot of people that will just not actually read an entire article and will maybe just read important parts and not the whole thing just so that you can get the CPD points.’ (Participant 9, Female, 25 years old)

## Discussion

Coronavirus disease 2019 has caused profound changes in the way that CPD activities are delivered, resulting in a paradigm shift towards online learning. This changed the way that HCPs, including young audiologists, engage in their ongoing professional training. However, some of these changes have created new challenges, hindering professional development. Despite the disruptive nature of the pandemic, it prompted the development of innovative approaches to CPD among HCPs. Literature identifies a need for online CPD courses to be well-designed considering HCP needs and be structured according to learning theories.^14^ To our knowledge, no study has been conducted within the South African context to understand how COVID-19 has transformed the CPD of young audiologists within the private sector.

Participants reported improved flexibility in accessing educational resources and networking opportunities online, at a time that suited them. Furthermore, the virtual platforms of CPD provided HCPs the advantage of convenience as it can now minimise disruptions to their clinical responsibilities. Such activities may have facilitated greater participation in CPD activities, as the online platform transcends geographical barriers and overcomes the high costs associated with in-person activities. This finding concurs with a Nigerian study that investigated the impact of COVID-19 on the CPD of physiotherapists,^4^ with many participants indicating that online learning was cost-effective and feasible for the implementation of their CPD. This is also in keeping with the findings from a study conducted in India that investigated the perceptions of postgraduate students on online learning during the COVID-19 pandemic.^15^ The study reported that online learning broke monotonous routines or repetitive learning patterns and made educational material easily accessible, which was also reported in this study.

Virtual communication removed location barriers, allowing online CPD programmes to offer opportunities for HCPs to connect with peers, experts and mentors worldwide. This fosters global collaboration, knowledge sharing and networking, which are essential for the professional growth and advancement of young audiologists. Participants indicated that online learning provided them with the platform to obtain real-time updates of advances within their field as they were able to access webinars and virtual conferences that featured sessions on timely topics or recent advancements. This was also seen in the Nigerian study as the physiotherapists joined international conferences which increased their awareness and knowledge.^4^ Furthermore, a study conducted in Ghana reported that the use of online methods of CPD enabled them to reach a larger number of HCPs when the dissemination of time-sensitive information is essential in comparison to traditional CPD methods such as in-person CPD activities.^16^

On the downside, the COVID-19 pandemic presented significant challenges to the CPD of young audiologists. The cancellation of in-person events deprived HCPs of valuable hands-on learning experiences and networking opportunities, which are crucial components of CPD for young audiologists. Moreover, the practical skills of these audiologists may have been compromised, as many of the participants graduated during the midst of the pandemic or were in the latter stages of completing their undergraduate training. Online platforms may not provide ample opportunities for networking and building professional relationships as expressed by many participants of this study. During face-to-face CPD, participants can engage in discussions and seek support from peers. However, in an online setting, this interaction is often limited, leading to a sense of isolation. A systematic review that looked into the effectiveness of distance learning strategies for allied health workers’ CPD highlighted the need for HCPs to have ‘time out’ to physically attend CPD courses.^3^ It is critical to understand that in-person training provides opportunities for young audiologists to observe demonstrations and practical exercises, which can be crucial for mastering skills that they are not very confident in.

Furthermore, while online CPD platforms offer flexibility, they can lack the interpersonal interaction provided by in-person training. This can affect engagement levels and the depth of learning achieved by HCPs. This is consistent with the research findings of an Indonesian study which found that online learning does not produce the same learning outcome as face-to-face learning. They concluded that for this reason, in-person CPD for HCPs is preferable in comparison to online learning, particularly for skills training.^17^ Furthermore, a research study that investigated the effectiveness of e-learning in comparison to conventional teaching among medical undergraduates during the COVID-19 pandemic highlighted that although online learning can be effective in supporting the educational process, it cannot replace the existing system of education.^18^ Therefore, hybrid learning should be considered by stakeholders of CPD. Hybrid learning, also known as blended learning, combines both face-to-face and online learning.^19^ Hybrid learning will enhance accessibility by allowing HCPs to access CPD activities remotely while still having the option for face-to-face interactions and hands-on experiences when appropriate. Hybrid learning offers flexibility to HCPs, allowing them to choose the learning mode that best suits their needs, whether through workplace activities, online learning or a combination of both.

As HCPs engage with CPD activities facilitated through online platforms, a pertinent dilemma arises, which is the balance between meeting compliance standards and promoting authentic learning. The convenience of online activities brings with it a challenge, as it is imperative to determine the pursuit of CPD to ensure that it does not merely become a tick-box exercise but remains a meaningful endeavour for knowledge enrichment and skill refinement. Participants witnessed many of their colleagues engaging in CPD simply to meet the statutory requirements rather than to genuinely enhance their knowledge and skills. In-person CPD activities uniquely offer immediate feedback to facilitators regarding the quality and impact of the CPD session on HCPs’ skill development. Active participation during these sessions allows facilitators to gauge effectiveness firsthand, ensuring meaningful contributions to professional growth that may not be as easily assessed in virtual settings.^20^ Therefore, CPD providers need to carefully evaluate the credibility of online CPD activities to ensure that the HCP is accurately receiving their CPD and not just engaging in CPD to meet statutory requirements. Online courses should therefore incorporate interactive elements like quizzes, case studies, simulations and discussions to actively engage HCPs. This approach not only enhances retention and application of knowledge but also aligns well with various types of CPD presentations suited to the specific learning outcomes of HCPs. Each format – whether online, in-person or hybrid – can cater effectively to different learning objectives, ensuring that the chosen method is well matched to the desired educational outcomes.

### Limitations

This study may not have fully accounted for contextual factors such as access to online resources or individual socioeconomic status which could influence participants’ experiences of CPD during the pandemic.

### Recommendations

Without a doubt, online CPD will continue post the COVID-19 pandemic, with creative solutions to the challenges identified needing to be found. There is a need for ongoing research on online CPD and learning methods that encourage active participation and engagement on this platform to ensure that CPD contributes meaningfully towards the career advancement of HCPs and provides high standards of care to patients.

## Conclusion

As the world transitions into a post-pandemic era, the lessons learned from COVID-19 are shaping CPD activities for HCPs. Educators, policymakers and healthcare institutions must integrate these lessons into ongoing CPD initiatives, equipping HCPs with essential knowledge, skills and resources to effectively navigate future challenges. This evolution necessitates policymakers adopting policies that ensure the accreditation and quality assurance of online CPD, preserving its credibility and effectiveness. Furthermore, healthcare and educational institutions play pivotal roles by providing the necessary resources and infrastructure for continuous learning and skill development. Employers are also crucial in supporting CPD through provisions like time, funding and recognition of professional growth. Meanwhile, CPD providers must align their offerings with current healthcare needs, delivering relevant content through diverse platforms to cater effectively to the diverse needs of HCPs.

## Appendix 1: Interview schedule

1. Tell me about your experience with continuing professional development.
2. How do you access continuing professional development (CPD) activities?
3. What are some of the changes you would like to see in CPD activities?

### Possible probing questions:

1.1. What are the reasons you engage with CPD? 1.2. How often do you engage in CPD? 1.3. Do CPD activities contribute meaningfully to your clinical practice? 1.4. How has engaging in CPD changed your practice?

2.1. What are the barriers you encounter in accessing CPD? 2.2. What types of CPD activities do you do?

3.1. Are you aware of how many points you require? 3.2. Do you know what you get points for?
